# Lipids and Lipid‐Lowering Drugs With Risk of Intervertebral Disc Degeneration: A Mendelian Randomization Study

**DOI:** 10.1155/genr/5378960

**Published:** 2026-07-29

**Authors:** Aochen Xu, Jiabin Yuan

**Affiliations:** ^1^ Department of Spine Surgery, Ningbo No. 6 Hospital, 1059 East Zhongshan Road, Ningbo Zhejiang, 315000, China, nbdlyy.com; ^2^ Department of Spine Surgery, Changhai Hospital, Navy Military Medical University, 168 Changhai Road, Shanghai 200433, China, chhospital.com.cn

## Abstract

This study examined whether circulating lipid traits and lipid‐lowering drug targets are causally associated with intervertebral disc degeneration (IVDD) using a Mendelian randomization (MR) framework. We analyzed summary‐level data from genome‐wide association studies (GWASs) covering apolipoproteins, lipid parameters, and IVDD. Univariable analysis revealed that inherently elevated high‐density lipoprotein cholesterol (HDL‐C) correlated with reduced IVDD susceptibility. By contrast, low‐density lipoprotein cholesterol (LDL‐C), triglycerides (TGs), apolipoprotein A‐I (Apoa‐I), and apolipoprotein B (Apob) did not remain significant after Bonferroni correction. Upon adjusting for body mass index (BMI) in a multivariable framework, the protective link between HDL‐C and IVDD held strong, whereas LDL‐C, TG, and Apob remained nonsignificant. Summary data–based MR and drug–target MR analyses further suggested a significant association between NPC1L1 gene expression and IVDD susceptibility. Overall, these results highlight the potential involvement of lipid pathways in disc degeneration and justify further biological and clinical investigation.

## 1. Introduction

Intervertebral disc degeneration (IVDD) represents a widespread, long‐term musculoskeletal condition predominantly affecting the aging population, creating a substantial clinical and socioeconomic burden [1, 2]. Its most common manifestation is low back pain, which affects a considerable proportion of the global population [3]. Although IVDD has been extensively studied, the mechanisms responsible for its initiation and progression remain incompletely understood. Current evidence indicates that genetic predisposition, immune dysregulation, apoptosis, aberrant mechanical stress, and local inflammatory activity all contribute to the degenerative process. As a result, metabolic disturbance and inflammatory signaling have become major areas of interest in IVDD research [4].

Hyperlipidemia is a common metabolic abnormality and is strongly associated with body mass index (BMI), which is independently established as a hazard for IVDD [5]. Even so, the extent to which cholesterol‐related traits directly influence susceptibility to IVDD remains unresolved. Evidence from an animal model of hyperlipidemia showed accelerated disc degeneration together with altered expression of degeneration‐associated molecules compared with normolipidemic controls [6]. In parallel, several observational studies have linked serum lipid abnormalities to IVDD, suggesting that elevated low‐density lipoprotein cholesterol (LDL‐C) and triglycerides (TGs) have the potential to promote atherosclerotic change, reduce nutrient supply and repair capacity within the disc, and thereby aggravate degeneration [7–9]. By contrast, other reports have suggested that BMI plays the dominant role, that high‐density lipoprotein cholesterol (HDL‐C) may be of secondary importance, or that the lipid–IVDD relationship is simply inconsistent [10]. Such discrepancies are difficult to resolve in conventional epidemiological studies because residual confounding, selection effects, and reverse causation cannot be fully excluded.

The Mendelian randomization (MR) approach offers a robust statistical method to establish causality when traditional observational designs face inherent biases [11]. Utilizing naturally occurring genetic sequence variations as instrumental variables (IVs), this technique capitalizes on the random assortment of alleles. Ideally, these genetic proxies must strongly predict the exposure of interest but are less likely to be distorted by environmental confounders or by disease processes occurring after birth [12]. A valid MR design therefore depends on three assumptions: the instruments are robustly associated with the exposure, remain completely unlinked to potential confounders, and influence the clinical outcome exclusively via the target exposure.

The present study was designed to determine the potential causal influence of systemic lipid profiles on the development of IVDD. We therefore applied MR using publicly available large‐scale genome‐wide association study (GWAS) datasets. Because previous observational analyses and MR studies have indicated that BMI may itself be causally related to IVDD, we performed an additional multivariable evaluation to isolate the distinct impacts of lipid parameters while controlling for BMI [13]. Furthermore, several prior studies have suggested that statin therapy may exert beneficial effects in disc degeneration [14–16]. To explore whether genetically proxied lipid‐lowering targets might be relevant to IVDD, we also performed drug–target MR using variants in or near HMGCR, PCSK9, NPC1L1, and APOB as proxies for statins, PCSK9 inhibitors, ezetimibe, and APOB inhibitors, respectively [17].

## 2. Materials and Methods

### 2.1. Study Design

**FIGURE 1:**
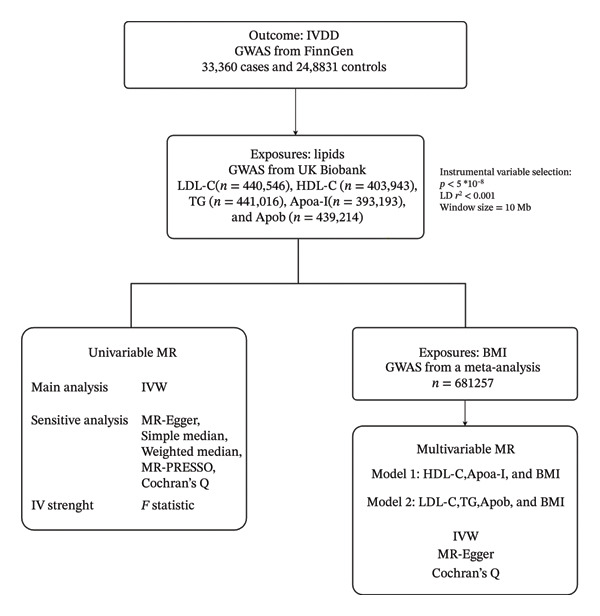
The study design. Instrumental variables (SNPs) were selected based on genome‐wide significance (*p* < 5∗10^−8^) and independence (linkage disequilibrium *r* ^2^ < 0.001, clumping window = 10 Mb). IVDD = intervertebral disc degeneration, GWAS = genome‐wide association study, MR = Mendelian randomization, BMI = body mass index, IVW = inverse‐variance weighted, HDL‐C = high‐density lipoprotein cholesterol, LDL‐C = low‐density lipoprotein cholesterol, TG = triglyceride, and Apo = apolipoprotein.

Figure 1 shows the overall analytical process. First, we carried out univariable MR analyses to test for correlation between circulating lipid and apolipoprotein traits (including HDL‐C, LDL‐C, TG, apolipoprotein A‐I [Apoa‐I], and apolipoprotein B [Apob]) and IVDD. We then established two multivariable MR models to account for possible pleiotropy and correlation among lipid‐related traits and to estimate their direct effects on the outcome [18]. Because BMI has been implicated as a causal determinant of IVDD, it was incorporated into the multivariable framework as a covariate.

We implemented the summary data–based Mendelian randomization (SMR) pipeline, merging GWAS outputs with expression quantitative trait locus (eQTL) datasets to pinpoint transcriptomic linkages. Lastly, targeted genetic modeling was executed to evaluate the therapeutic relevance of lipid‐lowering medications to IVDD. All the data included in this study were publicly accessible, and the relevant ethical approvals are detailed in the source publications.

### 2.2. Data Sources and Instrument Selection

#### 2.2.1. GWAS of IVDD

Summary statistics regarding IVDD were acquired via the FinnGen database. The analytical cohort consisted of 248,831 healthy reference individuals and 33,360 diagnosed subjects, classified according to the International Classification of Diseases framework (including ICD‐10 M51, ICD‐9 722, and ICD‐8 725 codes) (https://www.finngen.fi/en/access_results, accessed on 1 Dec 2023) [19].

#### 2.2.2. GWAS of Lipids and Apolipoproteins

Genetic data proxying circulating LDL‐C, HDL‐C, TG, Apoa‐I, and Apob were sourced from a major GWAS meta‐analysis restricted to European‐descent participants within the UK Biobank [20].

#### 2.2.3. GWAS of BMI

Genetic instruments for BMI were derived from the largest available meta‐analysis comprising 681,257 participants of European ancestry [21].

For the univariable MR analyses, independent single‐nucleotide polymorphisms (SNPs) linked to each respective trait were isolated using a strict significance boundary (*p* < 5 × 10^−8^), with linkage disequilibrium (LD) clumping thresholds of *r* ^2^ < 0.001 and a 10‐Mb window. In the multivariable models, significant proxies from all included traits were pooled and subsequently reevaluated with a 1000‐kb clustering window (*r* ^2^ < 0.001), systematically selecting the smallest *p* value across exposures. We also calculated the proportion of phenotypic variance explained by the selected SNPs and derived *F* statistics to evaluate instrument strength; *F* < 10 was considered indicative of weak instruments [22].

#### 2.2.4. eQTL Data

To proxy the four major lipid‐modifying pathways (HMGCR, PCSK9, NPC1L1, and APOB), we retrieved genetic instruments from publicly accessible cis‐eQTL databases. We sourced blood‐specific expression markers for HMGCR from the eQTLGen Consortium (accessed on Dec 2019). For PCSK9 expression in whole blood and the transcript levels of NPC1L1 and APOB in subcutaneous adipose tissue, we utilized summary statistics from the GTEx Consortium (V8 release, accessed on Jan 2018). The isolated variants were positioned within a ± 1‐Mb window of the corresponding gene probe and reached statistical significance for gene expression levels at a PeQTL threshold of less than 5 × 10^−8^.

For the LDL‐C–mediated target analyses, we further extracted SNPs within 100 kb upstream and downstream of HMGCR, PCSK9, NPC1L1, and APOB that were significantly associated with LDL cholesterol levels in UK Biobank (*p* < 5 × 10^−8^) and had low LD (*r* ^2^ < 0.3).

### 2.3. In Vitro Validation via Quantitative Real‐Time PCR (RT‐qPCR)

To experimentally validate the protective effect of HDL predicted by our MR analyses, human nucleus pulposus (NP) cells were cultured and subjected to lipotoxic stress. Cells were divided into four groups: control, oxidized low‐density lipoprotein (ox‐LDL) treatment, ox‐LDL + HDL cotreatment, and HDL alone. After the designated treatment period, total RNA was extracted using TRIzol reagent (Invitrogen) according to the manufacturer’s protocol. The expression levels of the catabolic marker MMP13 and the core ferroptosis regulator SLC7A11 were quantified using RT‐qPCR. Relative gene expression was calculated using the 2^−ΔΔCt method, with GAPDH serving as the internal reference.

## 3. MR Analyses

### 3.1. Univariable MR and Multivariable MR

The principal causal estimates were derived utilizing the inverse‐variance weighted (IVW) algorithm [23]. Depending on the heterogeneity results, either fixed‐effect or random‐effects IVW models were applied, with the random‐effects model used when heterogeneity was significant. To ensure robustness, supplementary assessments including the simple median, weighted median, and MR‐Egger techniques were executed. We relied on the MR‐Egger intercept and Cochran’s *Q* metric to, respectively, screen for directional pleiotropic bias and inter‐instrument variance [24, 25]. Additionally, the MR‐PRESSO framework was applied to spot and neutralize horizontal pleiotropy, alongside a leave‐one‐out validation procedure to confirm no solitary polymorphism exclusively dictated the results [26, 27]. Multivariable MR was then conducted using multivariable IVW as the primary estimator. Two models were prespecified: Model 1 included HDL‐C, Apoa‐I, and BMI, whereas Model 2 included LDL‐C, TG, Apob, and BMI. Multivariable MR‐Egger was additionally used to assess possible measured and unmeasured pleiotropy [28]. The rationale for splitting the multivariable MR into two distinct models was grounded in both biology and statistical robustness. Apoa‐I and Apob are the primary structural apolipoproteins of HDL and LDL, respectively. Including all lipid and apolipoprotein traits in a single comprehensive model would introduce severe multicollinearity, which can inflate standard errors and destabilize the multivariable IVW estimates. Grouping them by their corresponding biological lipoparticles effectively mitigated this issue.

### 3.2. SMR Analyses

The SMR pipeline was subsequently employed to assess the relationship linking IVDD with genetically simulated transcriptomic activity by fusing GWAS and eQTL data. To differentiate true pleiotropy from simple linkage, the heterogeneity in dependent instruments (HEIDI) evaluation was also incorporated. A *P*_*H* *E* *I* *D* *I*_ value < 0.05 was interpreted as evidence that the SMR signal might be driven by LD between distinct causal variants rather than by a common causal variant. These analyses were carried out using SMR software (Version 1.3.1, accessed on 20 May 2022) [29].

All statistical analyses were carried out in R (Version 4.2.3) using the TwoSampleMR package (Version 0.5.6) [30] together with the MendelianRandomization package (Version 0.7.0).

## 4. Results

### 4.1. Evaluation of Circulating Lipids and IVDD Risk via Univariable MR

**FIGURE 2:**
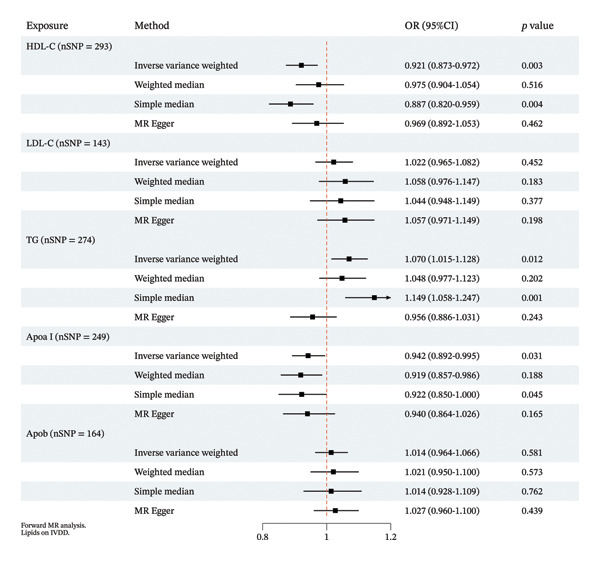
Univariable Mendelian randomization results using different methods. nSNP = number of single‐nucleotide polymorphisms, OR = odds ratio, CI = confidence interval, HDL‐C = high‐density lipoprotein cholesterol, LDL‐C = low‐density lipoprotein cholesterol, TG = triglyceride, Apo = apolipoprotein, and IVDD = intervertebral disc degeneration.

When the causal effects of the five lipid‐related traits on IVDD were evaluated, Cochran’s *Q* test indicated significant instrument heterogeneity (*p* < 0.05; Supporting Table S1), and we therefore prioritized the random‐effects IVW model. As shown in Figure 2, IVW estimates suggested associations between genetically predicted HDL‐C (OR = 0.921; 95% CI = 0.873–0.972; *p* = 0.003), Apoa‐I (OR = 0.942; 95% CI = 0.892–0.995; *p* = 0.031), and TG (OR = 1.067; 95% CI = 1.014–1.128; *p* = 0.0123) and IVDD risk. After Bonferroni correction (*p* = 0.05/5), only the association with HDL‐C remained significant. The weighted median, simple median, and MR‐Egger analyses yielded effect directions comparable to the IVW results. Except for TG (intercept = 0.005, *p* = 9.1*e* − 5), MR‐Egger did not indicate substantial horizontal pleiotropy. Removal of outliers identified by MR‐PRESSO did not materially alter the estimates. According to MR guidelines, this indicates that the nominal association seen in IVW was likely driven by pleiotropic, nonlipid pathways, further corroborating our conclusion that TG does not independently cause IVDD. No SNP had an F statistic below 10 (Supporting Table S2). Scatter plots and leave‐one‐out analyses (Supporting Figures S1A–E and S2A–E) did not suggest that any single SNP disproportionately drove the findings. Because heterogeneity was present, we next performed multivariable MR analyses.

### 4.2. Multivariable MR Analysis of Lipid‐Related Traits and BMI on IVDD

**FIGURE 3:**
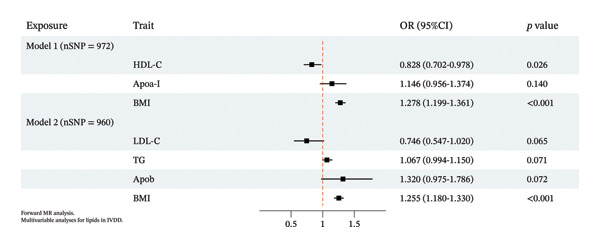
Multivariable Mendelian randomization results. nSNP = number of single‐nucleotide polymorphisms, OR = odds ratio, CI = confidence interval, HDL‐C = high‐density lipoprotein cholesterol, LDL‐C = low‐density lipoprotein cholesterol, TG = triglyceride, Apo = apolipoprotein, BMI = body mass index, and IVDD = intervertebral disc degeneration.

Given prior epidemiological evidence supporting a causal link connecting BMI to IVDD, we applied multivariable models to reduce confounding and account for pleiotropic pathways. A total of 972 SNPs were included in Model 1 and 960 SNPs in Model 2 (Supporting Tables S3 and S4). In Model 1, where HDL‐C, Apoa‐I, and BMI were evaluated simultaneously, higher HDL‐C remained associated with a lower risk of IVDD (Figure 3). Multivariable MR‐Egger did not indicate horizontal pleiotropy. Although Cochran’s *Q* test suggested residual heterogeneity, the multivariable MR‐Egger estimates were directionally consistent with the IVW results (Supporting Table S5).

In Model 2, which incorporated LDL‐C, TG, Apob, and BMI, none of the three lipid traits showed a significant independent association with IVDD. The MR‐Egger results were consistent with the primary analysis. As observed in Model 1, heterogeneity remained present, whereas evidence of horizontal pleiotropy was not detected.

### 4.3. SMR Analyses

After integrating cis‐eQTL information from eQTLGen and GTEx, we obtained 924, 24, 11, and 161 drug–target–related probes or genes for HMGCR, PCSK9, NPC1L1, and APOB, respectively. For the SMR analyses, the top cis‐associated SNP for each target gene was used as the IV to evaluate whether genetically predicted target–gene expression was associated with IVDD.

SMR analysis revealed a statistically valid link between NPC1L1 activity and IVDD risk (*β* = 0.0497; *p* = 0.028). The corresponding HEIDI test did not support linkage as the explanation for this association (*p* = 0.563 > 0.05) (Supporting Table S6).

To validate the precision of the MR‐Egger estimates for the 6 instruments proxying NPC1L1, we calculated the *I* ^2^ statistic, yielding a value of 0.9884, which satisfies the NOME assumption and confirms the reliability of the analysis.

### 4.4. Target‐Specific MR Analysis of LDL‐C Modulation and IVDD

**FIGURE 4:**
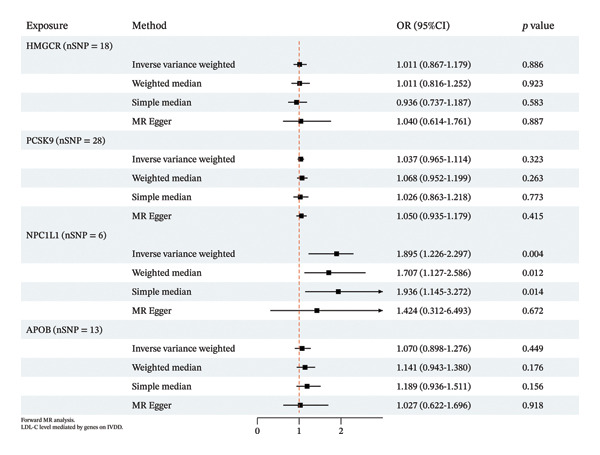
Drug–target Mendelian randomization results using different methods. nSNP = number of single‐nucleotide polymorphisms, OR = odds ratio, CI = confidence interval, and IVDD = intervertebral disc degeneration.

In our drug‐specific evaluations, we identified 18, 28, 6, and 13 genetic variants within or adjacent to HMGCR, PCSK9, NPC1L1, and APOB, respectively, using UK Biobank summary data for LDL‐C (Supporting Table S7). Drug–target MR results highlighted that only polymorphisms proximal to NPC1L1 (OR = 1.895; 95% CI = 1.351–2.657; *p* = 0.0002 < 0.0125) demonstrated a significant causal influence on IVDD risk through pathways linked to LDL‐C modulation (Figure 4 and Supporting Table S8). To explicitly clarify the directionality of these findings for clinical interpretation, higher genetically predicted NPC1L1 expression correlates with an increased risk of IVDD (OR > 1). This conversely indicates that inhibiting NPC1L1 activity, such as through the administration of ezetimibe, possesses a potential protective effect against disc degeneration. Conversely, LDL‐C–associated variants distal to the NPC1L1 region showed no association with the disease. This pattern suggests that the predicted benefit associated with ezetimibe may not be explained solely by lowering circulating LDL‐C. No evidence of horizontal pleiotropy or heterogeneity was found in these targeted models. Visual diagnostics, including scatter plots and leave‐one‐out results, are provided in Supporting Figures S3A–D and S4A–D.

### 4.5. In Vitro Validation of HDL‐Mediated Protection

**FIGURE 5:**
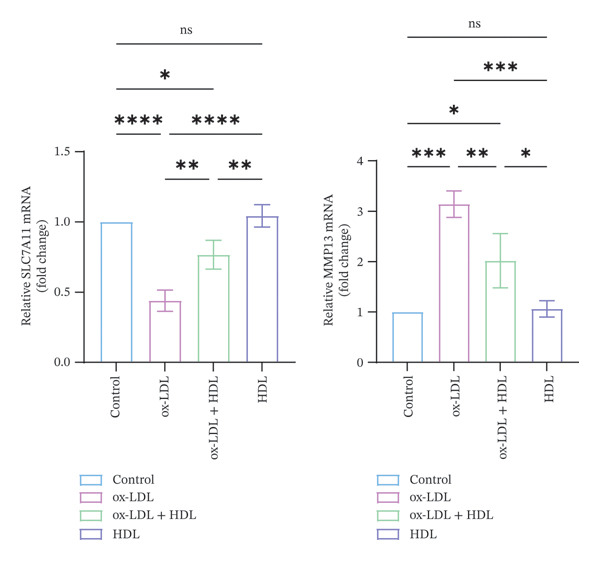
In vitro validation of the protective effect of HDL on human nucleus pulposus cells. RT‐qPCR analysis of the catabolic marker MMP13 and the ferroptosis regulator SLC7A11 in human NP cells treated with control, ox‐LDL, ox‐LDL + HDL, or HDL alone. Data are presented as mean ± SD from independent experiments. ns = not statistically significant; ∗: *p* < 0.05; ∗∗: *p* < 0.01; ∗∗∗: *p* < 0.001; ∗∗∗∗: *p* < 0.0001.

To bridge the gap between our systemic genetic proxies and local intervertebral disc biology, we performed an in vitro rescue experiment using human NP cells. Consistent with lipotoxicity‐induced degeneration, exposure to ox‐LDL significantly upregulated the expression of the catabolic marker MMP13 and downregulated the core ferroptosis defender SLC7A11 compared to the control group (*p* < 0.05). Crucially, cotreatment with HDL robustly rescued these pathological alterations, significantly suppressing MMP13 expression and restoring SLC7A11 levels (*p* < 0.05) (Figure 5). Treatment with HDL alone did not induce adverse effects compared to the control. These biological findings directly corroborate our computational MR results, confirming the local protective role of HDL against lipid‐induced disc degeneration.

## 5. Discussion

Our results provide genetic support for the hypothesis that higher genetically predicted HDL‐C levels are associated with a reduced risk of IVDD. This inference was reinforced by multivariable MR analyses in which other lipid‐related traits and BMI were considered simultaneously. Furthermore, to facilitate clinical interpretation, the causal estimates for HDL‐C were scaled from per‐standard deviation (SD) increments to standard clinical units. Assuming an approximate SD of 0.38 mmol/L for HDL‐C in the underlying UK Biobank cohort, a 1‐mmol/L increase in genetically predicted HDL‐C was associated with a decreased risk of IVDD (OR = 0.805; 95% CI = 0.700–0.928). This corresponds to an estimated 19.5% relative risk reduction per 1‐mmol/L increment.

Our findings are broadly aligned with previous clinical observations. A retrospective study published in 2022 involving 302 Chinese patients with IVDD found that HDL levels were inversely associated with disease severity after adjustment for age and BMI [10]. Likewise, Heuch et al. reported a negative relationship between HDL and low back pain prevalence in a large cross‐sectional study that included 33,692 Norwegian women and 30,031 men [31]. Similar trends were subsequently observed in a prospective cohort with 11 years of follow‐up [32]. Nevertheless, observational studies, even when large, cannot fully eliminate confounding or reverse causation, and some previous analyses did not include Apoa‐I and Apob simultaneously. By incorporating both apolipoprotein traits within an MR framework, the present study extends prior work and strengthens causal inference concerning lipid metabolism and IVDD.

The mechanistic relationship between serum lipid abnormalities and IVDD is still not fully understood. Intervertebral discs, which are composed of the nucleus pulposus, annulus fibrosus, and cartilaginous endplates, represent the most massive avascular tissues inside our anatomy [33]. Their nutritional maintenance depends largely on diffusion through the cartilaginous endplates [34]. Kurunlahti et al. reported that reduced nutrient diffusion within lumbar discs was closely linked to diminished lumbar arterial blood flow, a change that could impair disc repair and accelerate tissue damage [35]. Given that HDL‐C has antiatherosclerotic properties, facilitates reverse cholesterol transport, and may suppress lipid‐associated local inflammation, these biological effects may partly explain why genetically higher HDL‐C was related to a reduced risk of IVDD in our analyses. While impaired nutrient diffusion secondary to atherosclerosis has historically been proposed as a macroscopic mechanism linking dyslipidemia to IVDD, the field has increasingly shifted toward an immunometabolic microenvironmental perspective. Recent studies highlight the direct lipotoxic effects of abnormal lipid metabolites within the intervertebral disc [36]. Specifically, the accumulation of ox‐LDLs alongside toxic peroxides is known to trigger massive reactive oxygen species (ROS) generation. This oxidative burst catalyzes cellular aging and ferroptosis within the NP and cartilage endplate cells [37]. In this context, the genetically predicted protective effect of HDL‐C observed in our MR analysis may transcend systemic antiatherosclerotic functions; it likely also stems from HDL’s inherent antioxidant capacity to neutralize toxic lipid peroxides locally, thereby attenuating ferroptotic and senescent cascades and preserving the cellular integrity of the disc. Moving beyond our computational predictions, our in vitro RT‐qPCR experiments provide direct biological validation of this hypothesis. By demonstrating that HDL cotreatment effectively suppresses the lipotoxicity‐induced upregulation of MMP13 and rescues the expression of SLC7A11 in human NP cells, we confirm that HDL exerts profound local protective effects. This functional evidence tightly aligns with our MR findings, indicating that HDL not only acts systemically as an antiatherosclerotic agent but also actively preserves the immunometabolic and structural integrity of the intervertebral disc microenvironment.

In the univariable MR analyses, Apoa‐I showed a suggestive association with IVDD, but this signal was no longer apparent after multivariable adjustment. Apoa‐I is the main apolipoprotein found in HDL and plays a key role in its formation and metabolism [38]. Taken together, these outcomes imply that Apoa‐I itself might not independently determine IVDD risk once HDL‐C is accounted for, and that the apparent association is more likely to reflect broader HDL‐related biology. This attenuation indicates that the protective benefits of Apoa‐I are intrinsically dependent on overall HDL‐C levels. Consequently, for routine clinical risk assessment and prognostic monitoring of IVDD, standard HDL‐C panel testing appears sufficient, whereas additional screening for Apoa‐I may be redundant and is unlikely to yield independent clinical value.

Previous animal studies evaluating statins in IVDD have reported increased type II collagen expression and delayed degenerative progression after treatment [14, 39]. It is worth noting that in both our SMR and drug–target MR studies, HMGCR, which is a more widely used lipid‐lowering drug target, yielded negative results, whereas NPC1L1 showed a significant protective effect. Given the widespread clinical application of statins, this divergence warrants deeper consideration. Importantly, our mediation analysis revealed that the protective effect of NPC1L1 variants on IVDD was not mediated by a reduction in circulating LDL‐C levels, implying the involvement of lipid‐independent, pleiotropic pathways.

Several mechanistic differences may explain this discrepancy. First, while HMGCR governs hepatic cholesterol synthesis, NPC1L1 primarily regulates intestinal cholesterol absorption. Lifelong genetic downregulation of HMGCR might provoke compensatory mechanisms, such as the upregulation of intestinal cholesterol absorption, which could blunt its overall protective efficacy against IVDD. Second, beyond lipid lowering, NPC1L1 inhibition (ezetimibe) has been shown to exert potent systemic anti‐inflammatory and antioxidant effects, which are recognized as critical mitigating factors in the pathogenesis of disc degeneration. Conversely, although previous animal studies suggested that local administration of statins could delay disc degeneration, the lifelong, systemic genetic proxy of HMGCR inhibition might fail to achieve the requisite localized tissue concentrations within the highly avascular intervertebral disc to exert similar beneficial pleiotropic effects. Therefore, our findings suggest that NPC1L1 may represent a more specific and promising therapeutic target for IVDD, though further experimental studies are essential to elucidate its local molecular mechanisms in disc cellular metabolism.

From a practical standpoint, beyond primary prevention, optimizing serum lipid profiles may hold significant implications for postoperative prognosis in spine surgery. A critical challenge following spinal fusion or discectomy is the progressive development of adjacent segment disease (ASD) or recurrent disc herniation, which share similar degenerative pathophysiologies with primary IVDD. Given our causal inferences, aggressive postoperative lipid management could theoretically serve as a systemic adjunctive strategy to mitigate biomechanical and inflammatory stressors on adjacent segments, thereby delaying ASD onset. Future prospective clinical trials are warranted to evaluate the efficacy of lipid‐optimizing interventions in postoperative spinal cohorts.

This study has several notable strengths. First, we used a large and up‐to‐date IVDD GWAS dataset. Importantly, by sourcing exposure data from the UK Biobank and outcome data from the FinnGen consortium, we utilized two geographically and demographically distinct populations. This separation ensures minimal sample overlap, effectively satisfying a critical assumption of two‐sample MR and substantially minimizing weak instrument bias. Furthermore, we combined univariable MR, multivariable MR, SMR, and drug–target MR analyses, thereby improving the breadth of causal inference. Second, we performed multiple sensitivity analyses, including tests for heterogeneity and pleiotropy, MR‐PRESSO outlier correction, and F‐statistic evaluation of instrument strength. Third, by restricting analyses to datasets of European ancestry, we reduced the potential influence of population stratification. Fourth, while traditional observational studies are often plagued by reverse causality—such as IVDD‐induced chronic pain leading to sedentary behavior and secondary dyslipidemia—the MR framework intrinsically circumvents this bias. Because genetic variants are fixed at conception, acquired post‐disease lifestyle alterations cannot retrospectively modify the germline instruments used to proxy lipid levels, ensuring the directionality of our causal inferences.

Several limitations should also be considered. Heterogeneity could not be entirely eliminated in every analysis and may have affected the precision of some estimates. In addition, all GWAS data were derived from individuals of European ancestry. Given that the genetic architecture and environmental risk factors of disc degeneration may vary considerably across different ethnicities, the estimated effects represent an ancestry‐specific average. Therefore, explicitly expanding these genetic instruments to diverse, non‐European cohorts is essential before broad clinical translation can be generalized. MR can support causal inference but does not by itself reveal the underlying biological mechanisms, which still require experimental validation. Finally, the interpretation of drug–target MR findings should remain cautious for the reasons discussed above.

## 6. Conclusion

Overall, this study offers genetic evidence that indicates an inverse relationship between HDL‐C levels and the risk of IVDD. The SMR and drug–target MR analyses also highlight NPC1L1 as a potentially relevant pathway in IVDD susceptibility. Further mechanistic and clinical investigations are required to confirm these biological pathways and clarify the translational significance.

## Author Contributions

Aochen Xu: conceptualization, methodology, software, formal analysis, investigation, data curation, visualization, and writing–original draft. Jiabin Yuan: conceptualization, resources, supervision, project administration, validation, and writing–review and editing.

## Funding

No specific funding was received for this study.

## Conflicts of Interest

The authors declare no conflicts of interest.

## Supporting Information

Additional supporting information can be found online in the Supporting Information section.

## Supporting information


**Supporting Information** The Supporting material for this article can be found online and contains the following items. Supporting Tables (S1–S8): Detailed summary statistics and datasets for the Mendelian randomization (MR) frameworks. These include the primary causal effect estimates of circulating lipid traits on intervertebral disc degeneration (IVDD) via univariable MR (Table S1), multivariable MR models adjusting for body mass index (Tables S3–S5), and summary data–based MR (SMR) analysis (Table S6). The complete lists, genomic positions, and statistical characteristics (including beta, standard error, *p* value, and *F*‐statistics) of the genetic instruments (SNPs) used across all analyses and lipid‐lowering drug targets (HMGCR, PCSK9, NPC1L1, and APOB) are fully documented in Tables S2, S4, S7, and S8. Supporting Figures (S1–S4): Methodological validation and sensitivity analysis plots. Figures S1 and S3 display individual scatter plots illustrating the association between single‐nucleotide polymorphism (SNP) effect sizes on exposure traits (overall lipid parameters and specific drug–target–mediated LDL‐C levels, respectively) against their effects on IVDD susceptibility. Figures S2 and S4 provide corresponding leave‐one‐out permutation analysis curves to confirm the stability of the causal estimates and rule out the disproportionate influence of single outlier variants.

## Data Availability

The data that support the findings of this study are available in the supporting information of this article.
