# Supplementary material for: Lipids and Lipid‐Lowering Drugs With Risk of Intervertebral Disc Degeneration: A Mendelian Randomization Study
Source: Genet Res (Camb). 2026 Jul 29;2026:5378960. doi: 10.1155/genr/5378960 (PMC13420274; doi:10.1155/genr/5378960)

**Supplementary Figures**

**Supplementary Figure S1A:** Scatter plot showing the association of the SNP effects on HDL-C level against the SNP effects on Intervertebral Disc Degeneration;

**Supplementary Figure S1B:** Scatter plot showing the association of the SNP effects on LDL-C level against the SNP effects on Intervertebral Disc Degeneration;

**Supplementary Figure S1C:** Scatter plot showing the association of the SNP effects on TG levels against the SNP effects on Intervertebral Disc Degeneration;

**Supplementary Figure S1D:** Scatter plot showing the association of the SNP effects on Apoa-I level against the SNP effects on Intervertebral Disc Degeneration;

**Supplementary Figure S1E:** Scatter plot showing the association of the SNP effects on the Apob level against the SNP effects on Intervertebral Disc Degeneration;

**Supplementary Figure S2A:** Leave-one-out permutation analysis of the causal association between HDL-C level and Intervertebral Disc Degeneration;

**Supplementary Figure S2B:** Leave-one-out permutation analysis of the causal association between LDL-C level and Intervertebral Disc Degeneration;

**Supplementary Figure S2C:** Leave-one-out permutation analysis of the causal association between TG level and Intervertebral Disc Degeneration;

**Supplementary Figure S2D: Leave-one-out permutation analysis of the causal** association between Apoa-I Intervertebral Disc Degeneration;

**Supplementary Figure S2E:** Leave-one-out permutation analysis of the causal association between Apob level and Intervertebral Disc Degeneration;

**Supplementary Figure S3A:** Scatter plot showing the association of the SNP effects on LDL-C level mediated by the HMGCR gene against the SNP effects on Intervertebral Disc Degeneration;

**Supplementary Figure S3B:** Scatter plot showing the association of the SNP effects on LDL-C level mediated by the PCSK9 gene against the SNP effects on Intervertebral Disc Degeneration;

**Supplementary Figure S3C:** Scatter plot showing the association of the SNP effects on LDL-C level mediated by the NPC1L1 gene against the SNP effects on Intervertebral Disc Degeneration;

**Supplementary Figure S3D:** Scatter plot showing the association of the SNP effects on LDL-C level mediated by the APOB gene against the SNP effects on Intervertebral Disc Degeneration;

**Supplementary Figure S4A:** Leave-one-out permutation analysis of the causal association between LDL-C level mediated by the HMGCR gene and Intervertebral Disc Degeneration;

**Supplementary Figure S4B:** Leave-one-out permutation analysis of the causal association between LDL-C level mediated by the PCSK9 gene and Intervertebral Disc Degeneration;

**Supplementary Figure S4C:** Leave-one-out permutation analysis of the causal association between LDL-C level mediated by the NPC1L1 gene and Intervertebral Disc Degeneration;

**Supplementary Figure S4D: Leave-one-out permutation analysis of the causal** association between LDL-C level mediated by the APOB gene and Intervertebral Disc Degeneration;

**Supplementary Figure S1A.** Scatter plot showing the association of the SNP effects on HDL-C level against the SNP effects on Intervertebral Disc Degeneration
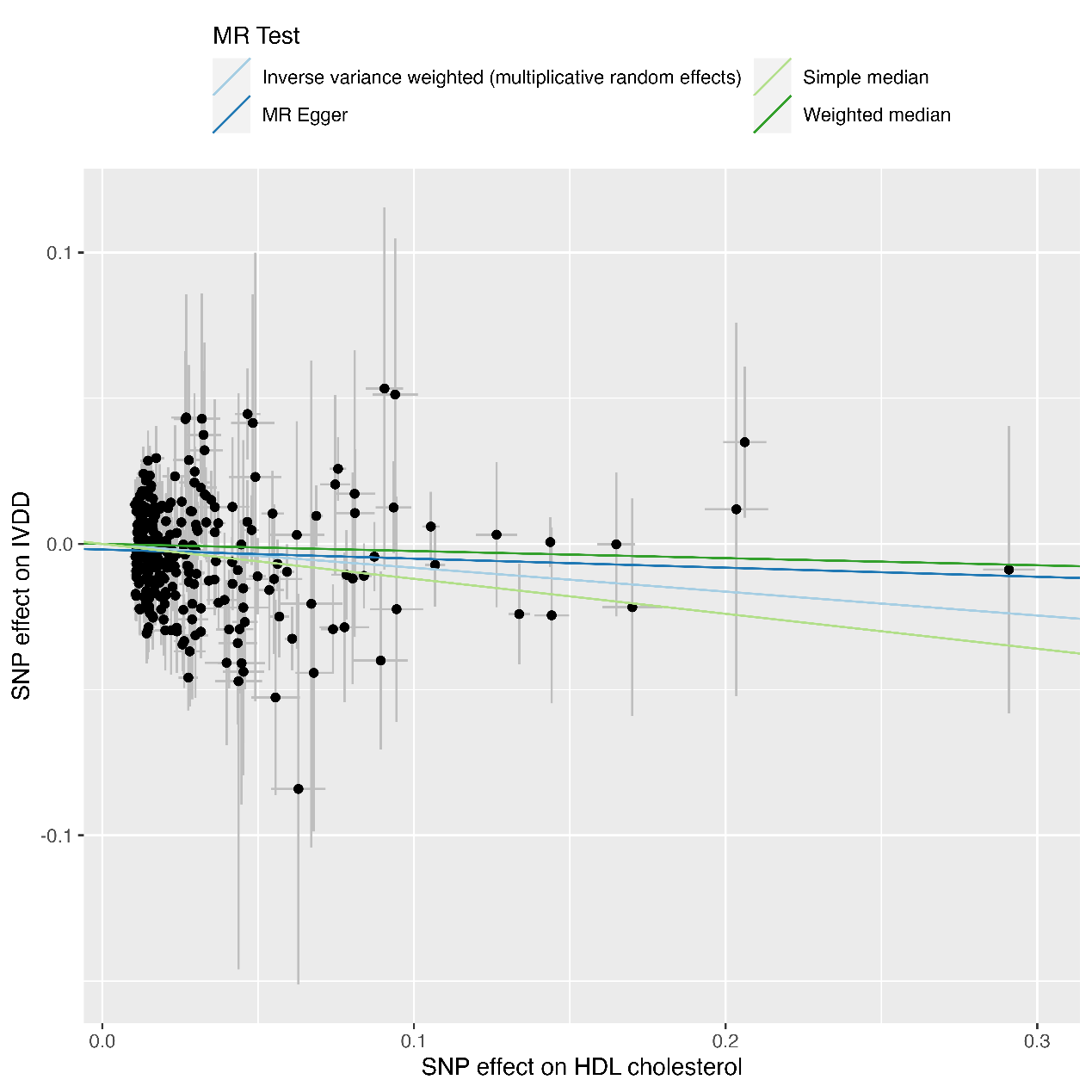


**Supplementary Figure S1B:** Scatter plot showing the association of the SNP effects on LDL-C level against the SNP effects on Intervertebral Disc Degeneration
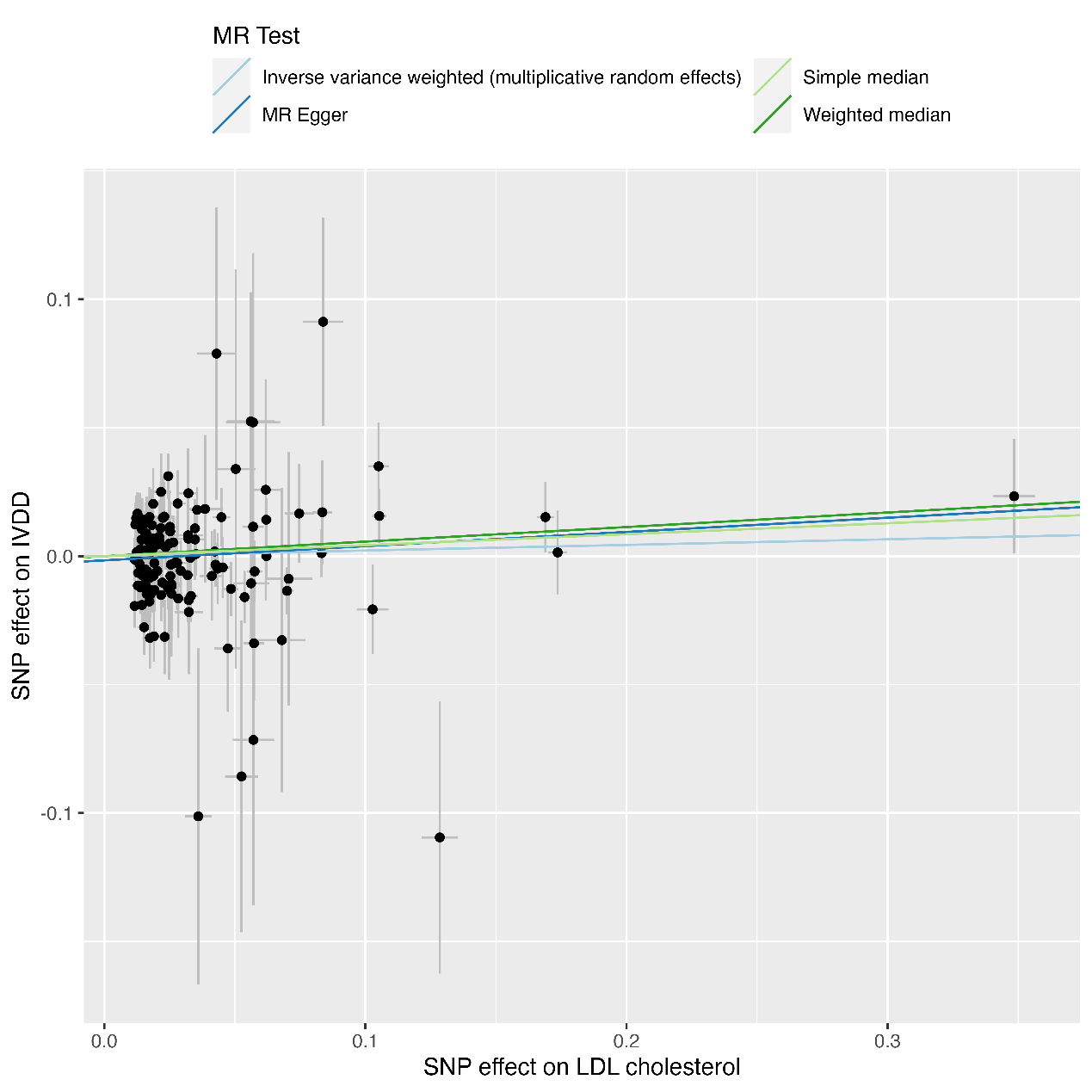


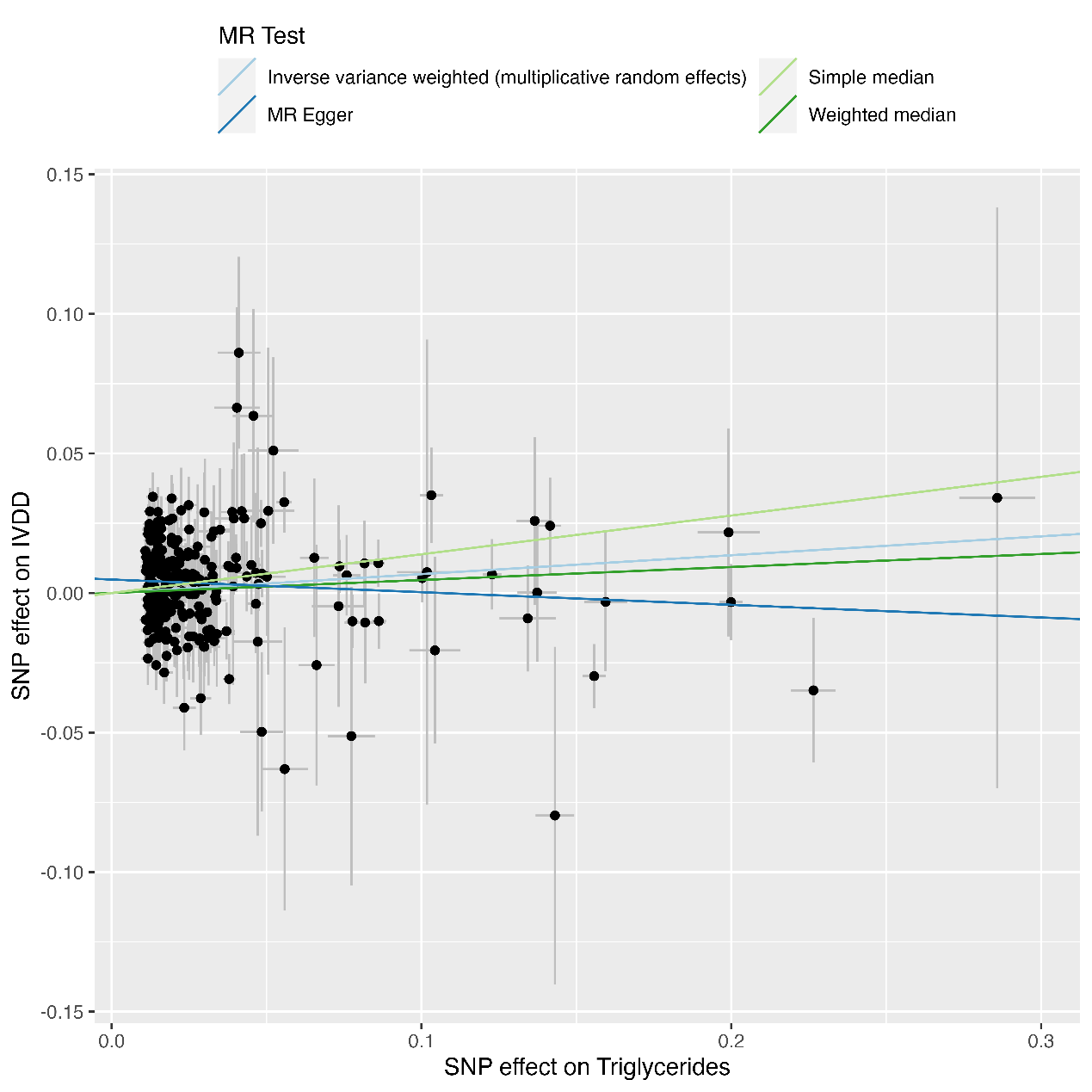
**Supplementary Figure S1C: Scatter plot showing the association of the SNP effects on TG levels against the SNP effects on Intervertebral Disc Degeneration**


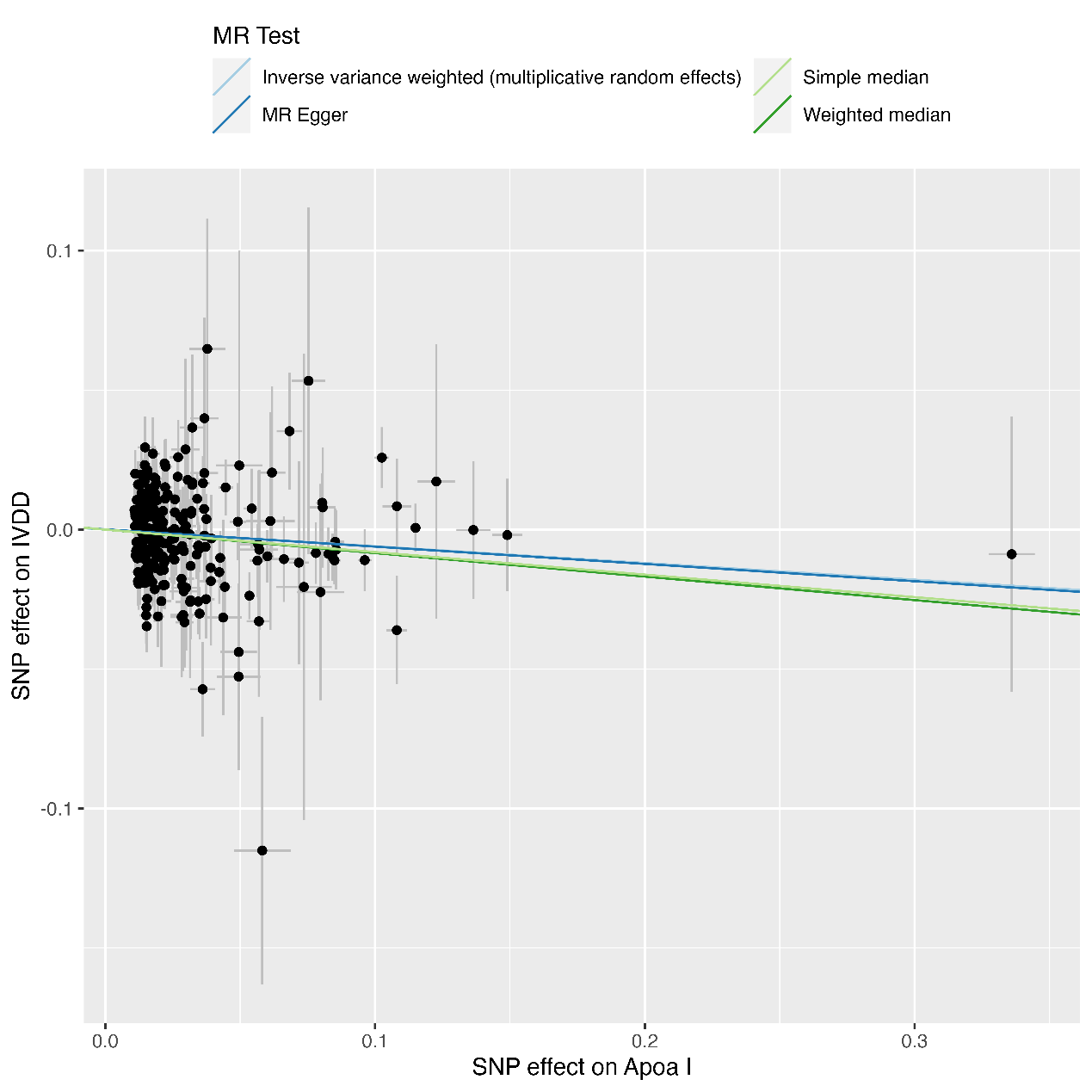


**Supplementary Figure S1D:** Scatter plot showing the association of the SNP effects on Apoa I levels against the SNP effects on Intervertebral Disc Degeneration

**Supplementary Figure S1E:** Scatter plot showing the association of the SNP effects on Apob levels against the SNP effects on Intervertebral Disc Degeneration
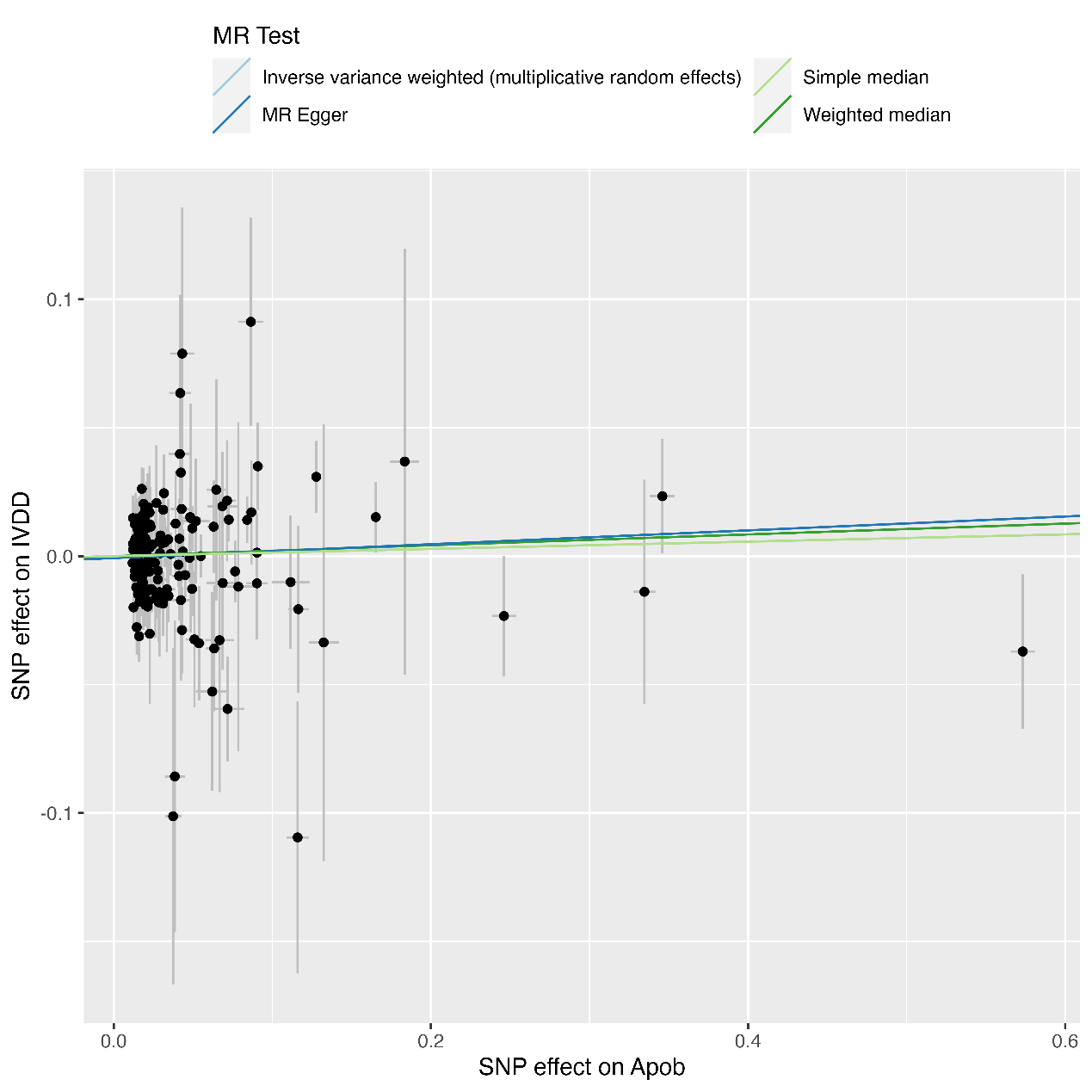


**Supplementary Figure S2A:** Leave-one-out permutation analysis of the causal association between HDL-C level and Intervertebral Disc Degeneration
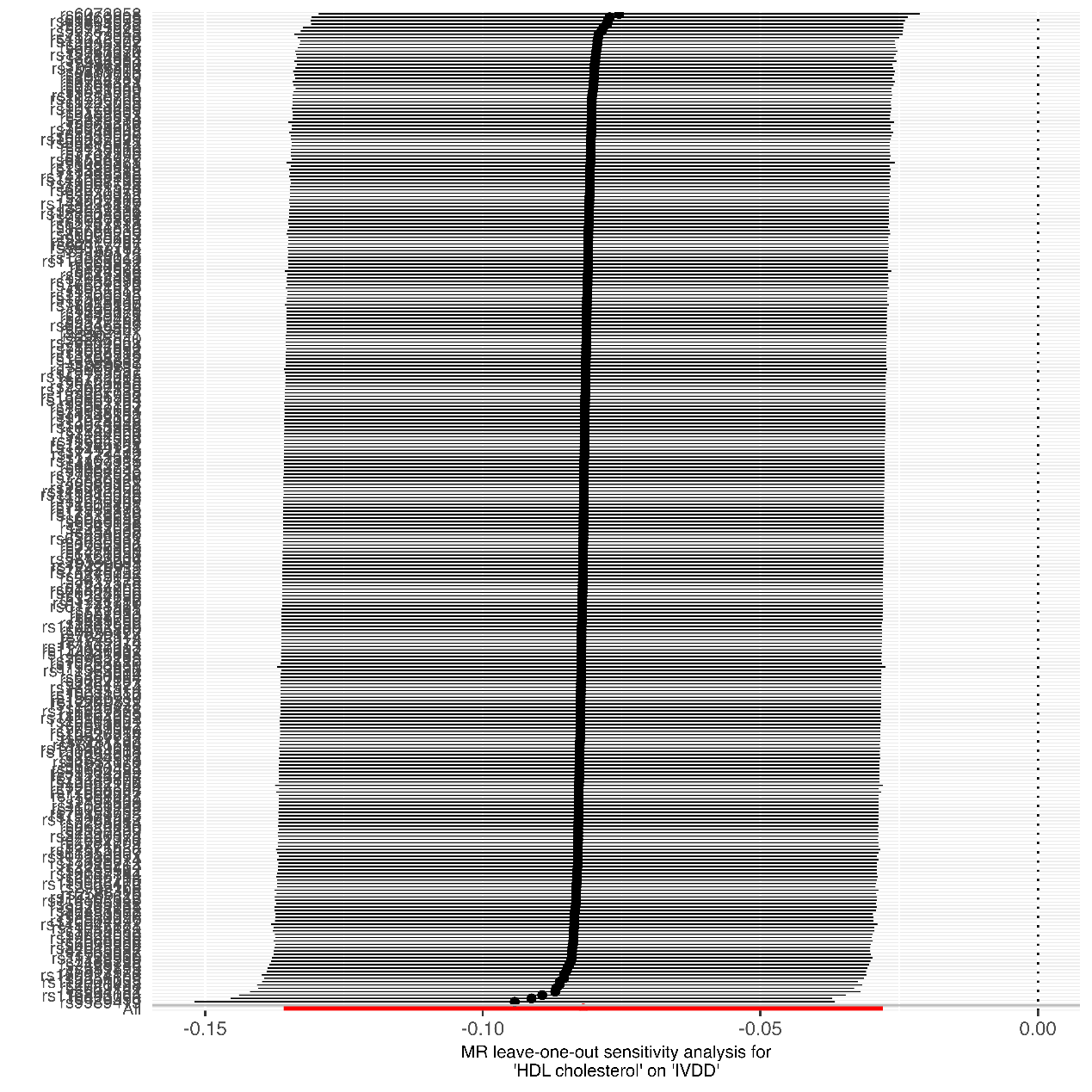


**Supplementary Figure S2B:** Leave-one-out permutation analysis of the causal association between LDL-C level and Intervertebral Disc Degeneration
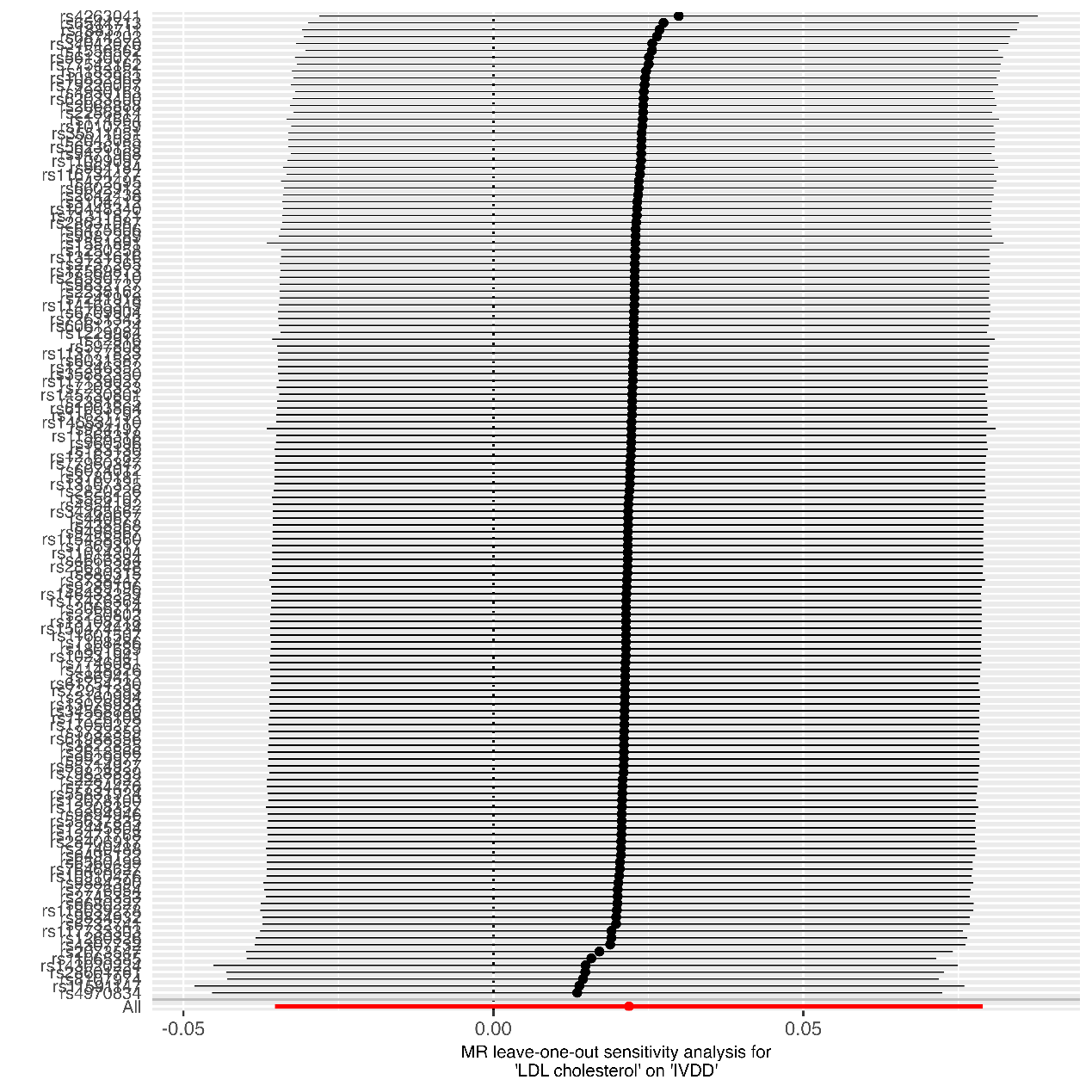


**Supplementary Figure S2C:** Leave-one-out permutation analysis of the causal association between TG level and Intervertebral Disc Degeneration
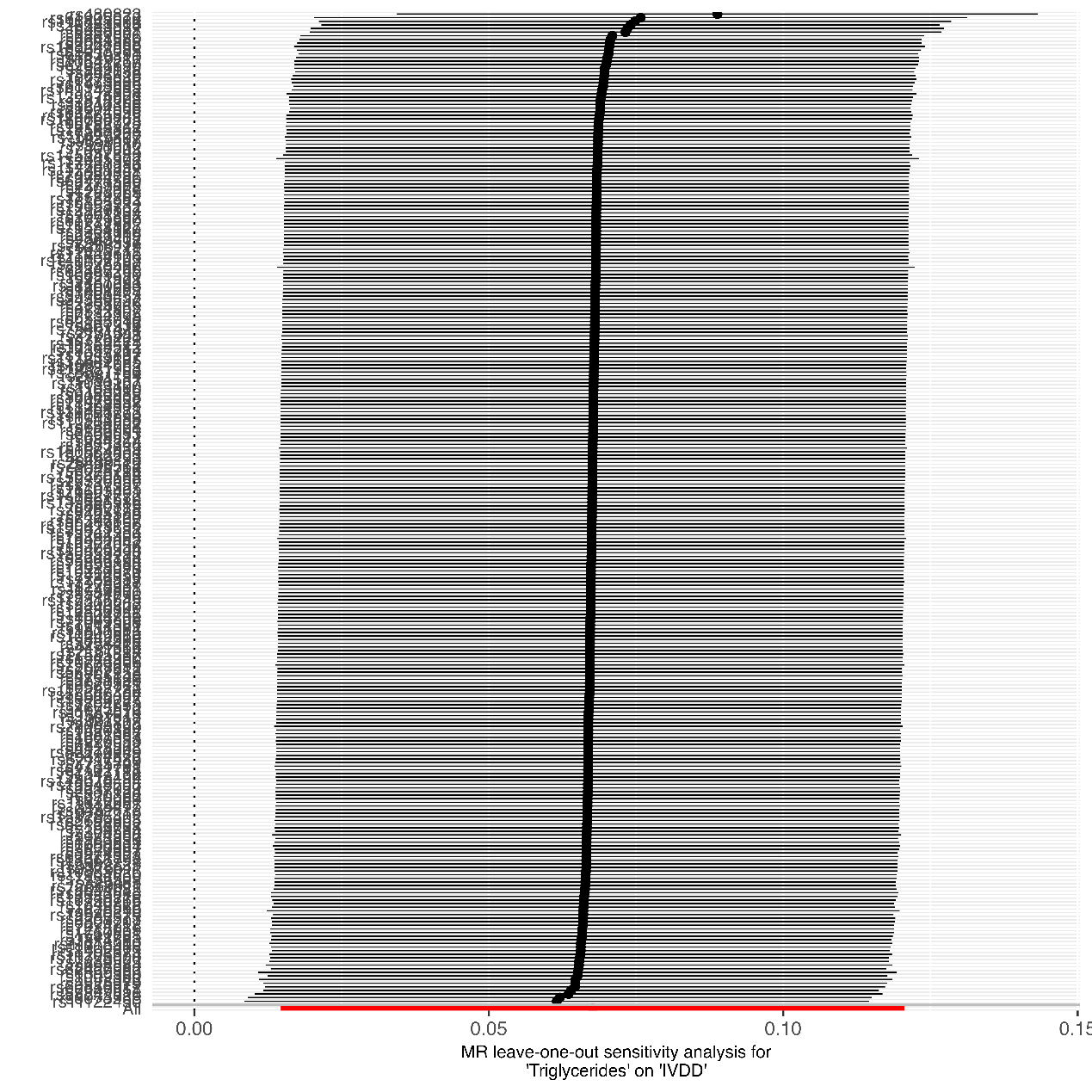


**Supplementary Figure S2D:** Leave-one-out permutation analysis of the causal association between Apoa I level and Intervertebral Disc Degeneration
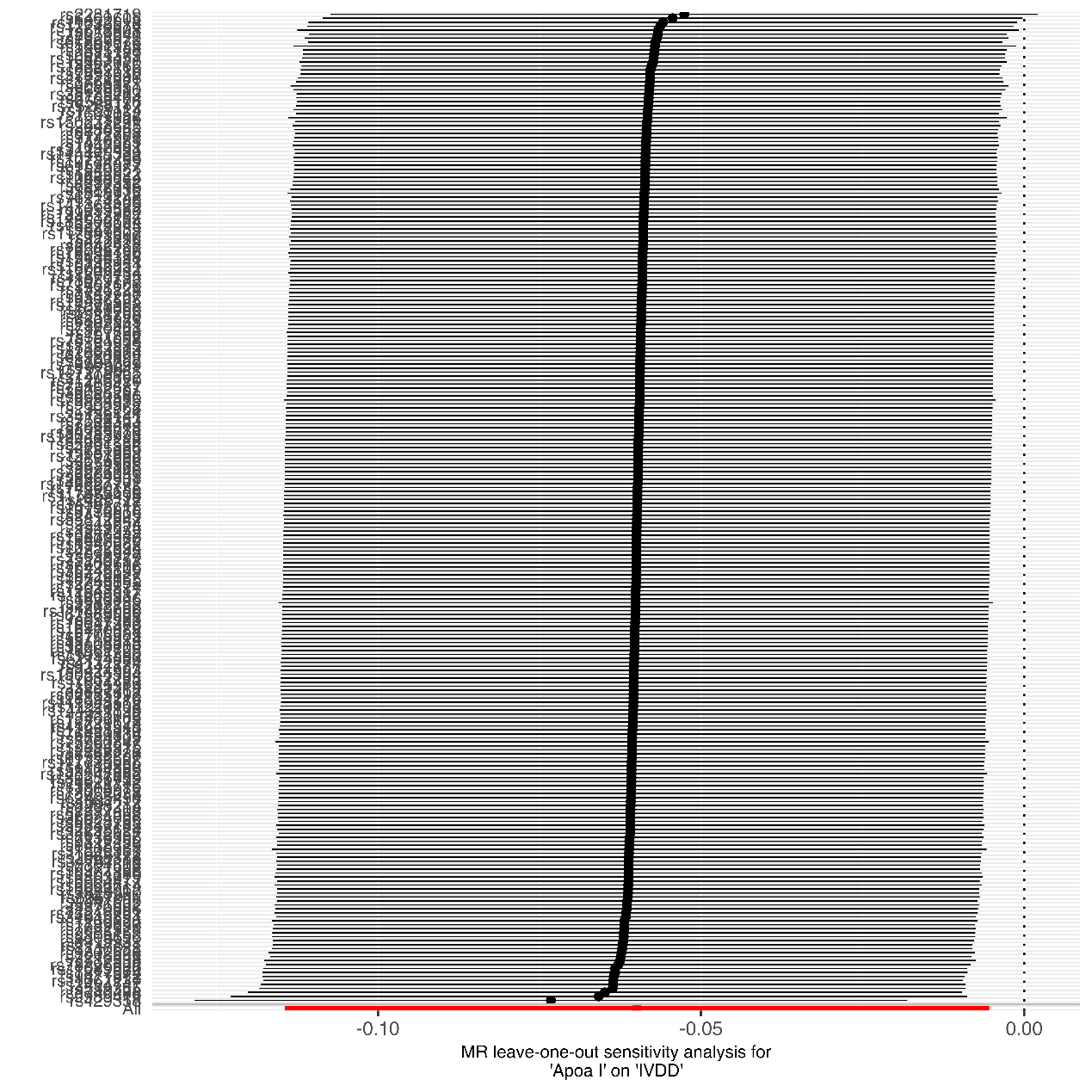


**Supplementary Figure S2E:** Leave-one-out permutation analysis of the causal association between Apob level and Intervertebral Disc Degeneration
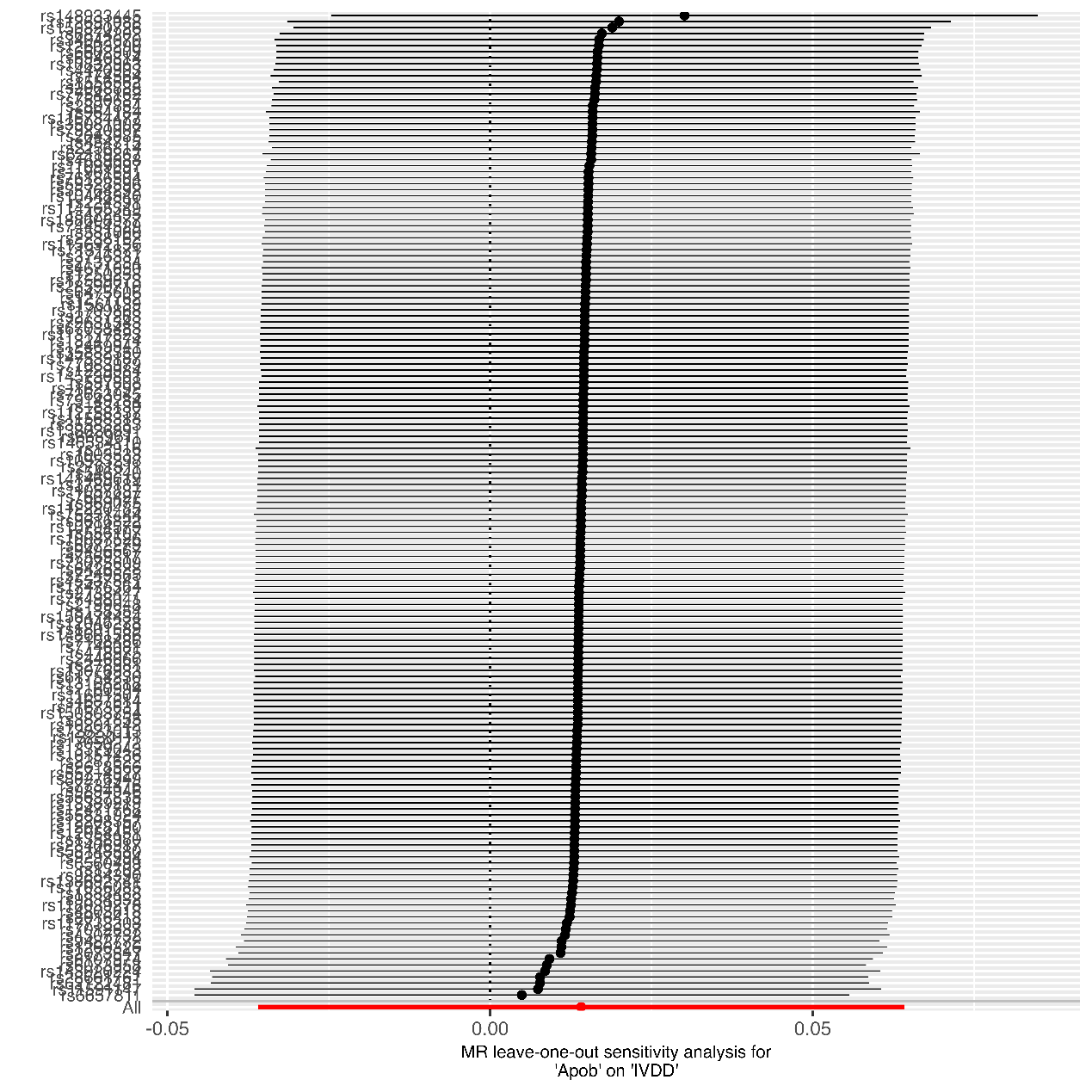


**Supplementary Figure S3A:** Scatter plot showing the association of the SNP effects on LDL-C level mediated by the HMGCR gene against the SNP effects on Intervertebral Disc Degeneration
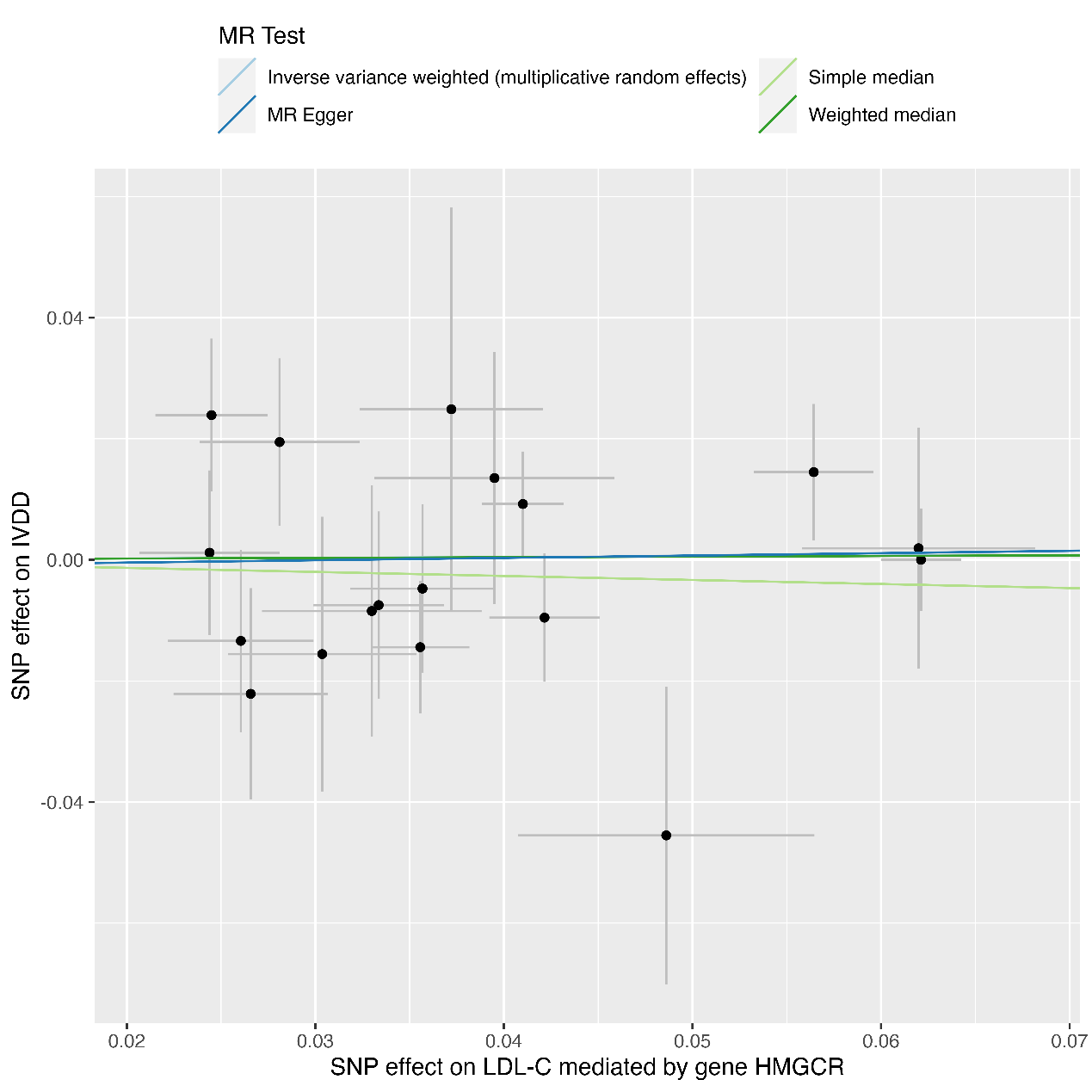


**Supplementary Figure S3B:** Scatter plot showing the association of the SNP effects on LDL-C level mediated by the PCSK9 gene against the SNP effects on Intervertebral Disc Degeneration
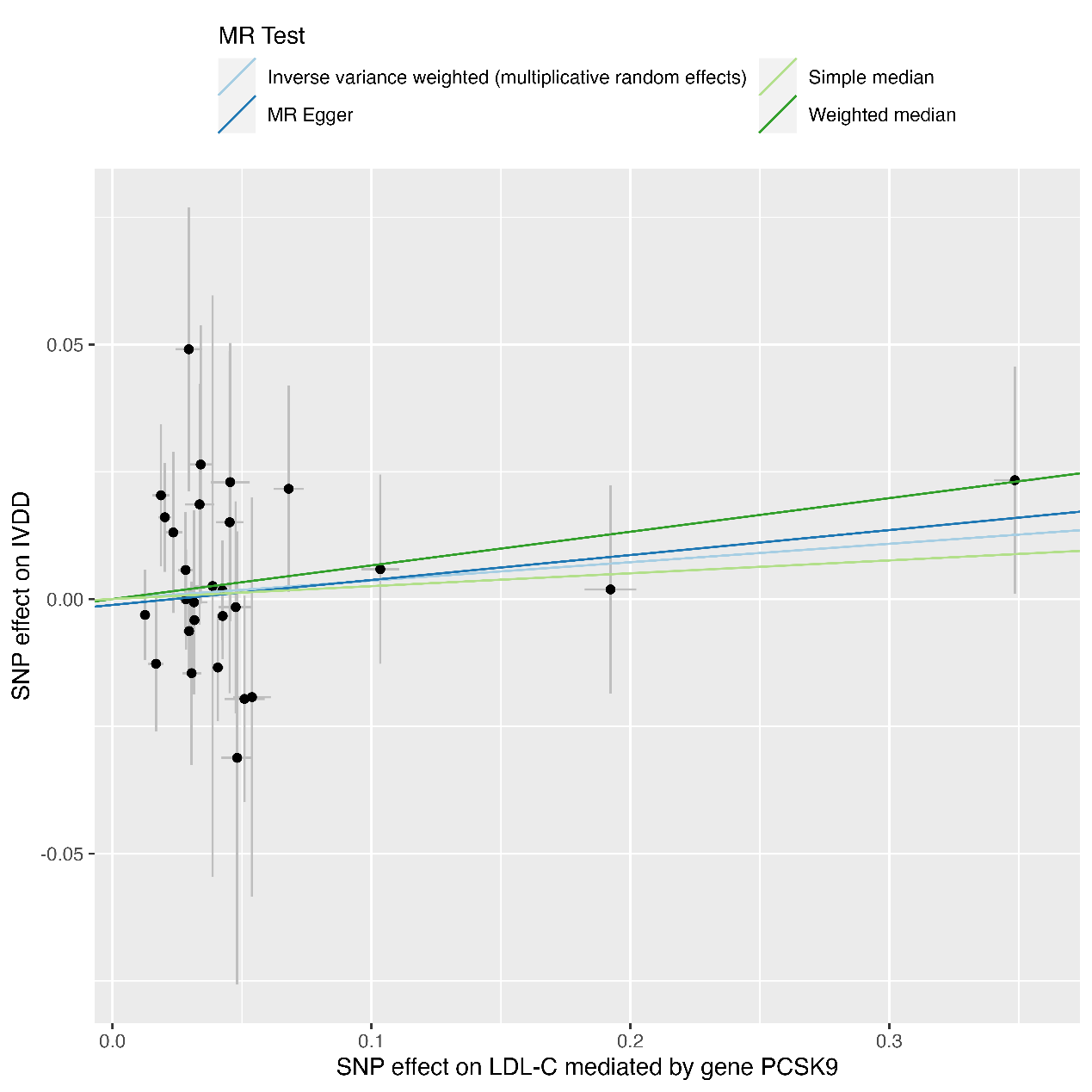


**Supplementary Figure S3C:** Scatter plot showing the association of the SNP effects on LDL-C level mediated by the NPC1L1 gene against the SNP effects on Intervertebral Disc Degeneration
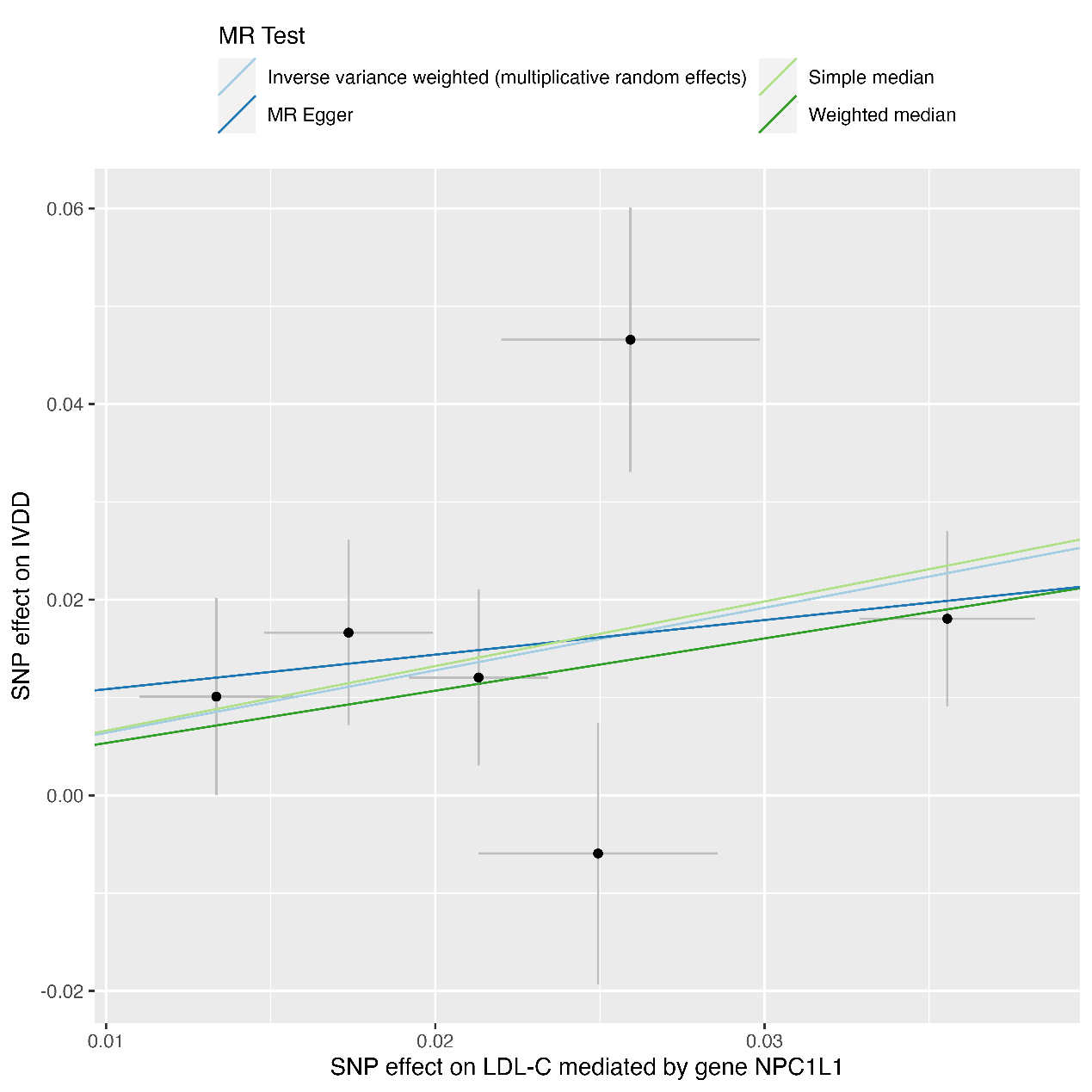


**Supplementary Figure S3D:** Scatter plot showing the association of the SNP effects on LDL-C level mediated by the APOB gene against the SNP effects on Intervertebral Disc Degeneration
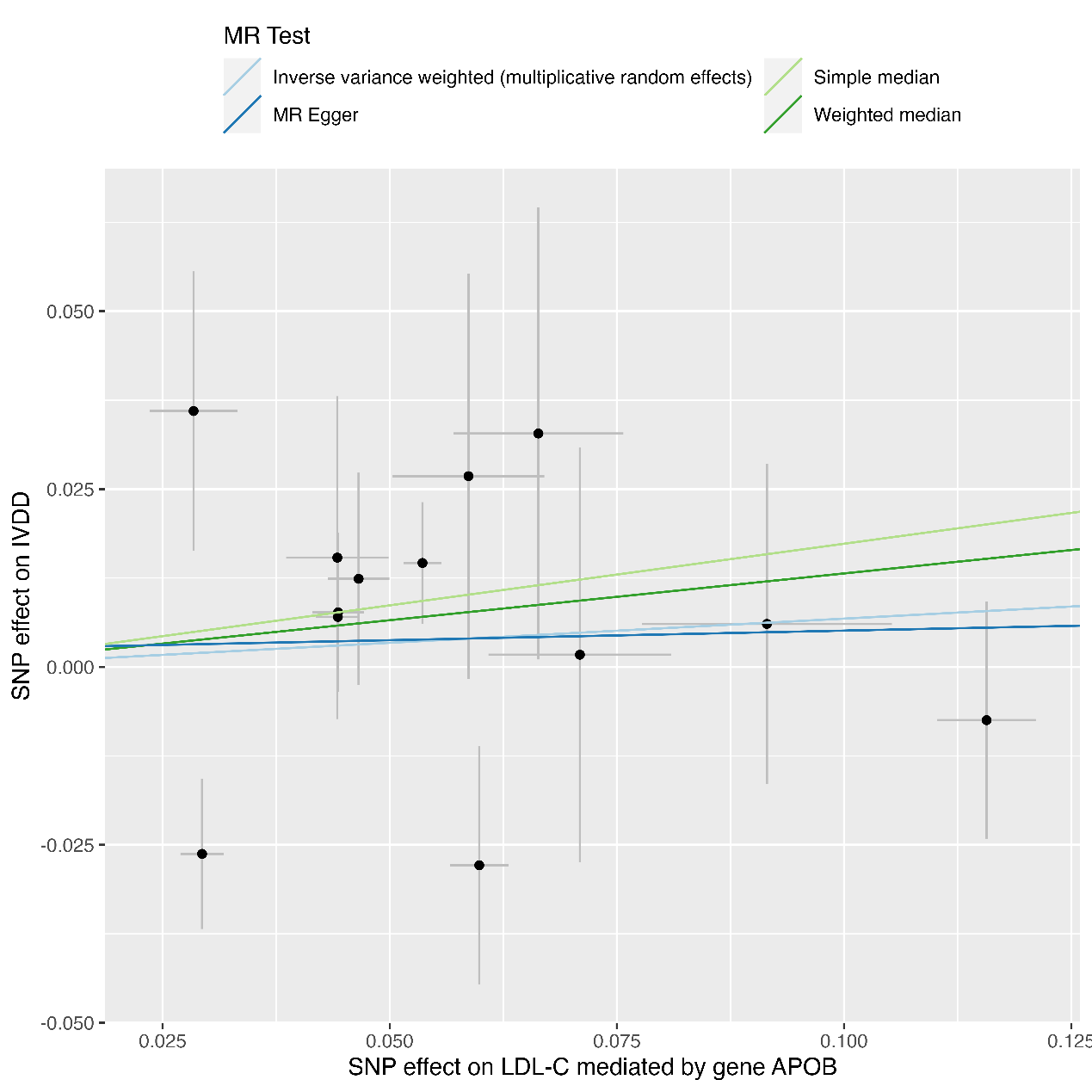


**Supplementary Figure S4A:** Leave-one-out permutation analysis of the causal association between LDL-C level mediated by the HMGCR gene and Intervertebral Disc Degeneration
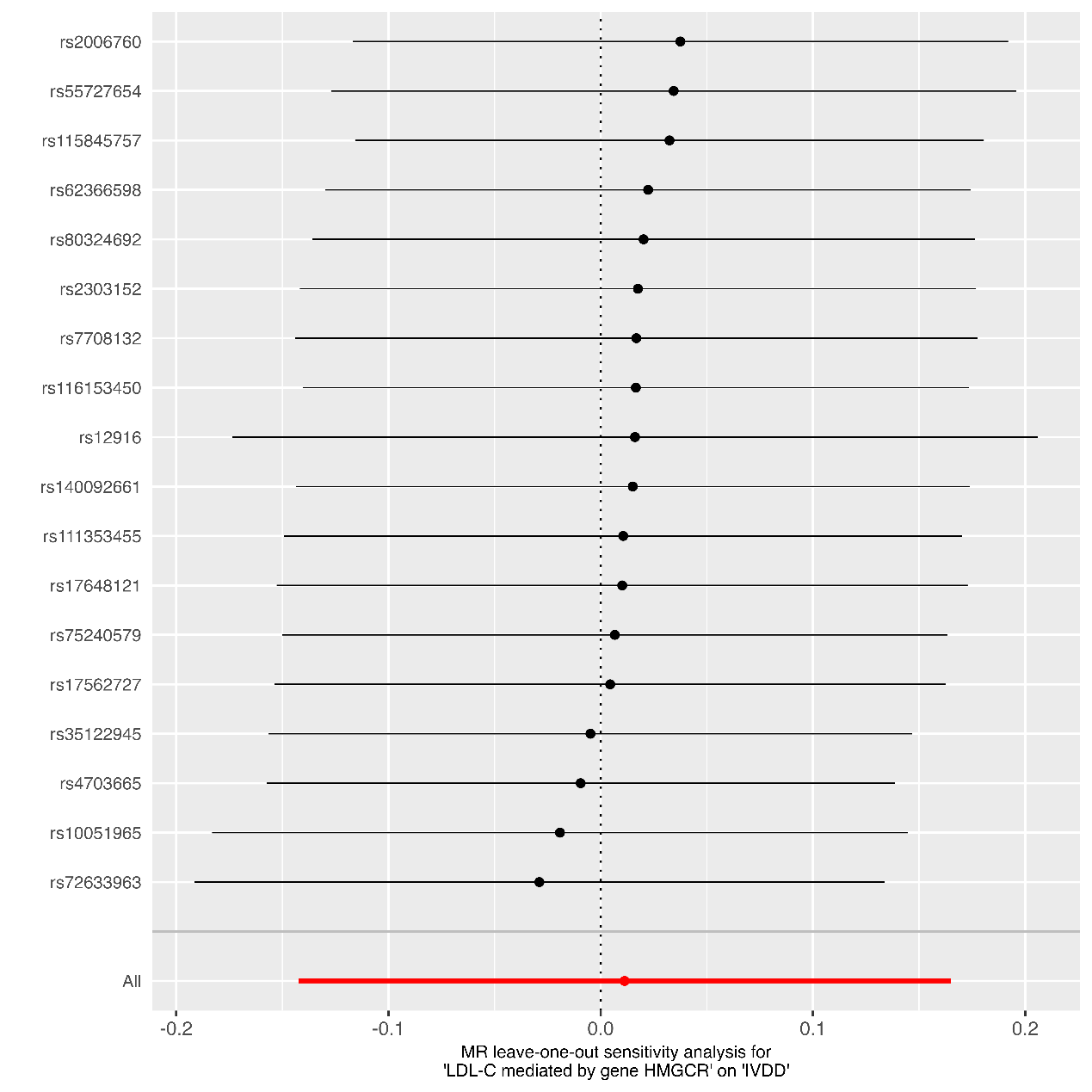


**Supplementary Figure S4B:** Leave-one-out permutation analysis of the causal association between LDL-C level mediated by the PCSK9 gene and Intervertebral Disc Degeneration
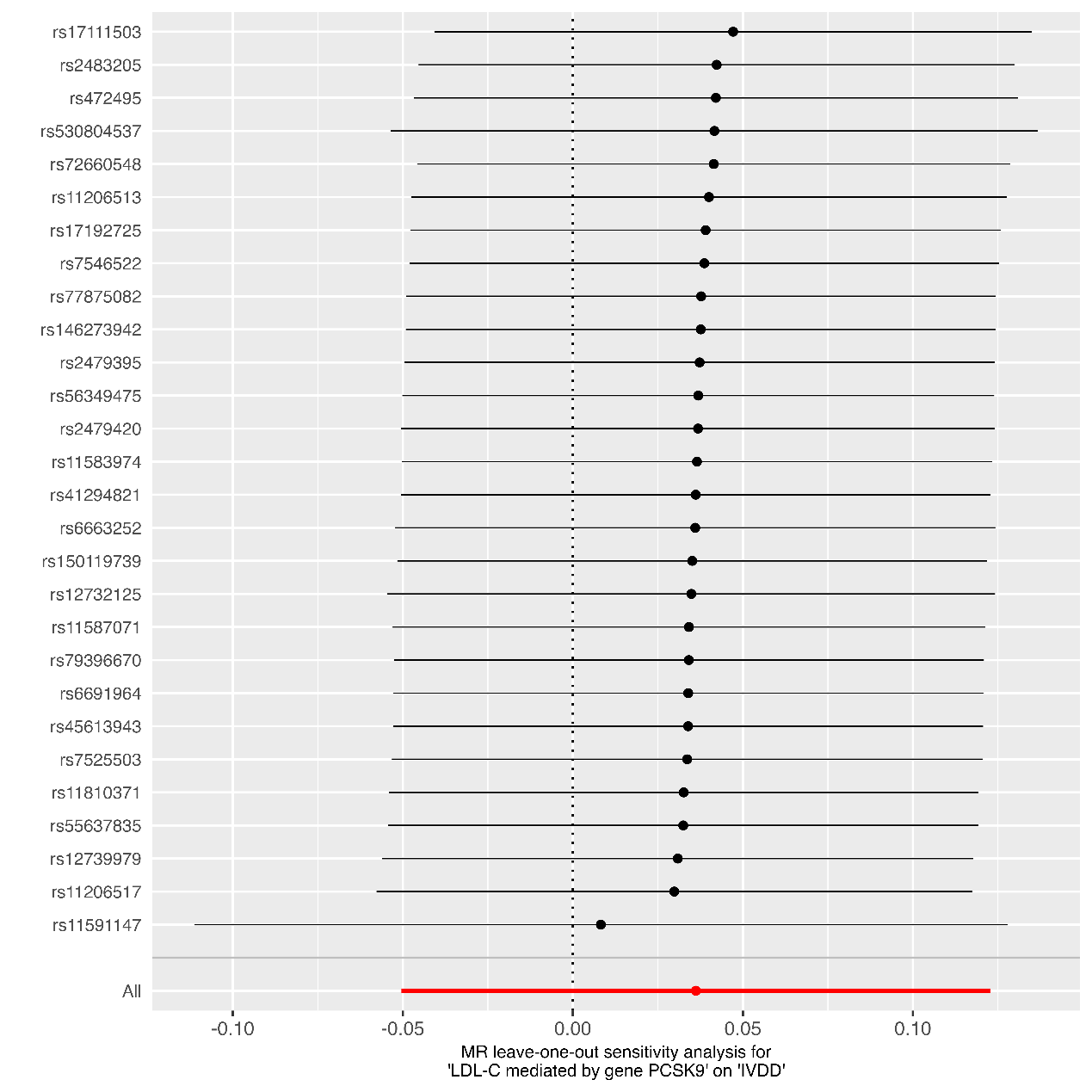


**Supplementary Figure S4C:** Leave-one-out permutation analysis of the causal association between LDL-C level mediated by the NPC1L1 gene and Intervertebral Disc Degeneration
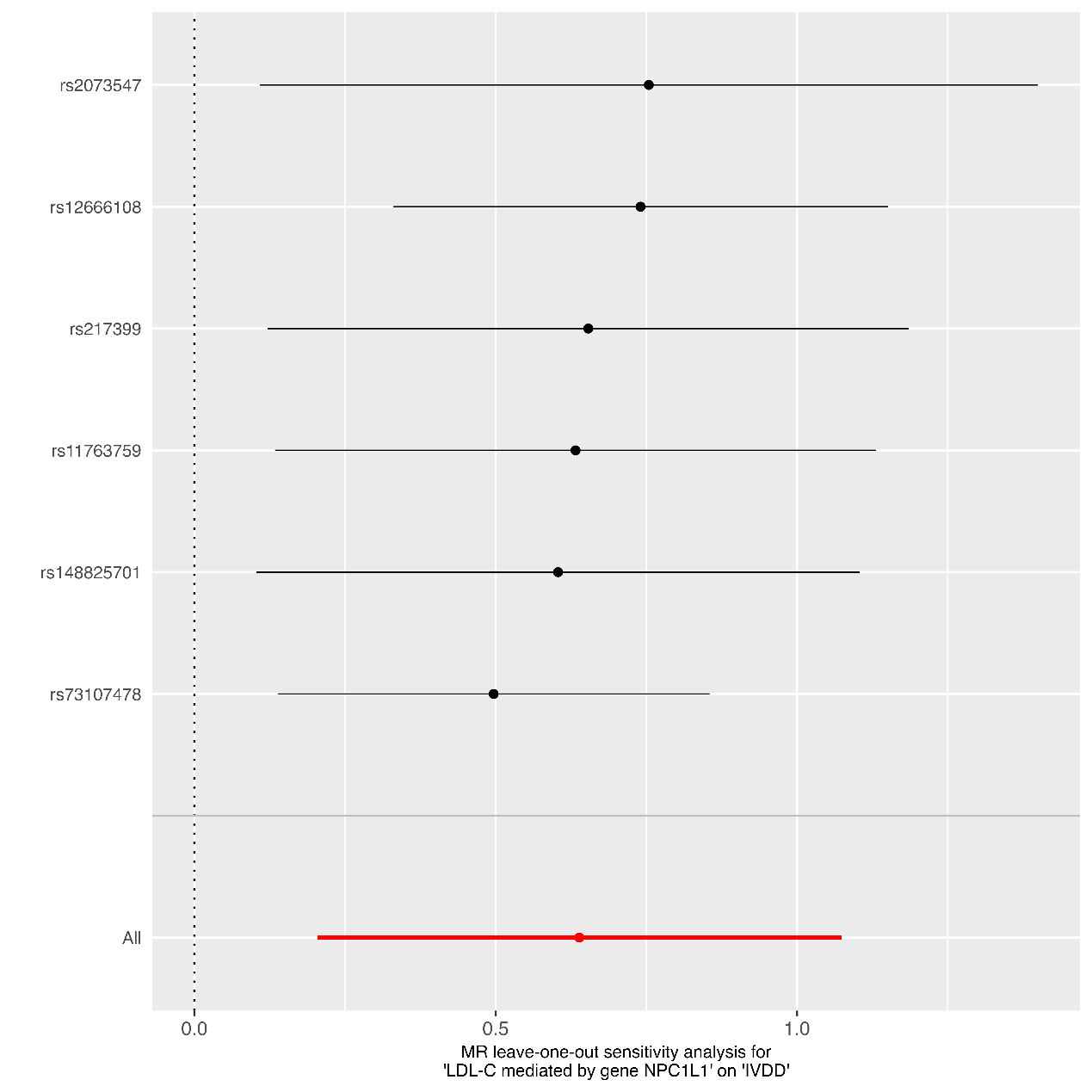


**Supplementary Figure S4D:** Leave-one-out permutation analysis of the causal association between LDL-C level mediated by the APOB gene and Intervertebral Disc Degeneration
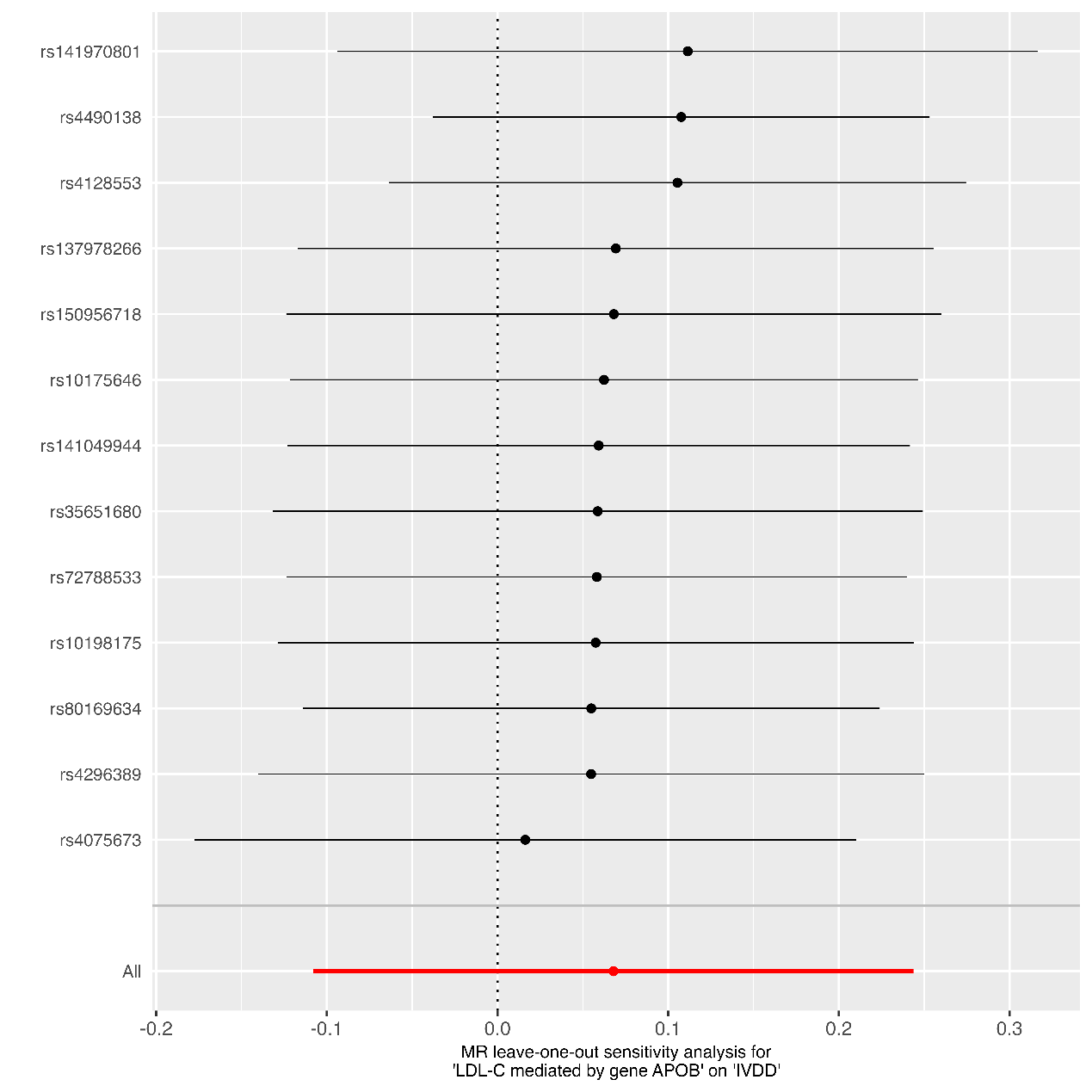

Supplement: Supplementary file 1 — Supporting Information The Supporting material for this article can be found online and contains the following items. Supporting Tables (S1–S8): Detailed summary statistics and datasets for the Mendelian randomization (MR) frameworks. These include the primary causal effect estimates of circulating lipid traits on intervertebral disc degeneration (IVDD) via univariable MR (Table S1), multivariable MR models adjusting for body mass index (Tables S3–S5), and summary data–based MR (SMR) analysis (Table S6). The complete lists, genomic positions, and statistical characteristics (including beta, standard error, p value, and F‐statistics) of the genetic instruments (SNPs) used across all analyses and lipid‐lowering drug targets (HMGCR, PCSK9, NPC1L1, and APOB) are fully documented in Tables S2, S4, S7, and S8. Supporting Figures (S1–S4): Methodological validation and sensitivity analysis plots. Figures S1 and S3 display individual scatter plots illustrating the association between single‐nucleotide polymorphism (SNP) effect sizes on exposure traits (overall lipid parameters and specific drug–target–mediated LDL‐C levels, respectively) against their effects on IVDD susceptibility. Figures S2 and S4 provide corresponding leave‐one‐out permutation analysis curves to confirm the stability of the causal estimates and rule out the disproportionate influence of single outlier variants. [file GENR-2026-5378960-s001.zip › suplementary figure.docx]
